# Innovative technologies and social inequalities in health: A scoping review of the literature

**DOI:** 10.1371/journal.pone.0195447

**Published:** 2018-04-03

**Authors:** Daniel Weiss, Håvard T. Rydland, Emil Øversveen, Magnus Rom Jensen, Solvor Solhaug, Steinar Krokstad

**Affiliations:** 1 Department of Public Health and Nursing, Norwegian University of Science and Technology, Trondheim, Norway; 2 HUNT Research Center, Department of Public Health and Nursing, Norwegian University of Science and Technology, Levanger, Norway; 3 Department of Sociology and Political Science, Norwegian University of Science and Technology, Trondheim, Norway; 4 Library Section for Humanities, Education and Social Sciences, Norwegian University of Science and Technology, Trondheim, Norway; 5 Levanger Hospital, Nord-Trøndelag Hospital Trust, Levanger, Norway; Universita degli Studi di Firenze, ITALY

## Abstract

The aim of this study was to systematically review the range, nature, and extent of current research activity exploring the influence of innovative health-related technologies on social inequalities in health, with specific focus on a deeper understanding of the variables used to measure this connection and the pathways leading to the (re)production of inequalities. A review process was conducted, based on scoping review techniques, searching literature published from January 1, 1996 to November 25, 2016 using MEDLINE, Scopus, and ISI web of science. Search, sorting, and data extraction processes were conducted by a team of researchers and experts using a dynamic, reflexive examination process. Of 4139 studies collected from the search process, a total of 33 were included in the final analysis. Results of this study include the classification of technologies based on how these technologies are accessed and used by end users. In addition to the factors and mechanisms that influence unequal *access* to technologies, the results of this study highlight the importance of variations in *use* that importantly shape social inequalities in health. Additionally, focus on health care services technologies must be accompanied by investigating emerging technologies influencing healthy lifestyle, genomics, and personalized devices in health. Findings also suggest that choosing one measure of social position over another has important implications for the interpretation of research results. Furthermore, understanding the pathways through which various innovative health technologies reduce or (re)produce social inequalities in health is context dependent. In order to better understand social inequalities in health, these contextual variations draw attention to the need for critical distinctions between technologies based on how these various technologies are accessed and used. The results of this study provide a comprehensive starting point for future research to further investigate how innovative technologies may influence the unequal distribution of health as a human right.

## Introduction

Despite expectations to the contrary, social inequalities in health appear to be increasing in many of the world’s most developed countries during an era of rapid innovative technological development [1–3]. As the quantification of health in modern society intensifies and innovative health technologies become the cornerstone of this transition, the connection between technology and health is garnering increased attention [4–7]. Recent years have witnessed an era of intensified technology use in health care services [8] as well as developments in personalized medicine and the use of big data for health purposes. These advances have promoted a growing dependency on technology in society and the collection of advanced information, including that of the personal genome, which are then used to influence the decisions and behaviors of not just ordinary citizens but also health personnel, private companies, and large institutions [9–11]. These innovations are generally seen as positive developments, improving the diagnostics and treatment of disease as well as general public health, however their wider societal implications can be questioned [10, 12–14]. It appears likely that these technologies could be improving general public health but at the cost of increasing inequalities in health [13, 15].

Various publications have addressed the importance of further investigating the potential implications that the rapid development and increased prioritization of various technological innovations in health have on the health of society as a whole [3, 10–12, 16, 17]. Other studies have empirically investigated the production of inequalities in health due to the advent of innovative technologies [18–20]. These studies demonstrate that individuals of higher socioeconomic status (SES) are the first to adopt, and benefit most from, the introduction of innovative technologies in health, creating social inequalities in health where they were once very low or nonexistent, or in some cases even inverting these inequalities (where improved health outcomes have moved from lower SES groups to higher SES groups). This phenomenon is further illustrated by results demonstrating larger social inequalities in health among populations suffering from illnesses for which effective preventive or treatment techniques have been developed [21]. These studies provide a starting point for investigating additional mechanisms that may explain the (re)production of social inequalities in health [22, 23]. As the rate of innovative health technology intensifies, a better understanding of this perspective is becoming increasingly important.

Still missing from the literature is a broad foundation from which to further investigate and explain the connection between technological innovations and social inequalities in health. The following questions are still in need of clarification:

- How are *innovative health technologies* defined in a social inequalities context?
- What are the implications of using various measurements of social inequality?
- How do existing studies explain the potential relationship between *innovative* health technology and social inequalities in health?
- How may *innovative* health technologies reduce or (re)produce social inequalities in health?

The aim of this study, therefore, was to systematically review the range, nature, and extent of current research activity exploring the influence of innovative health technologies on social inequalities in health, with specific focus on a deeper understanding of the variables used to measure this connection and the pathways leading to its (re)production.

## Methods

A systematic search process was conducted, based on scoping review techniques, [24, 25] for literature published from January 1, 1996 to November 25, 2016 using the following databases: MEDLINE, Scopus, and ISI web of science. The search was updated on November 25, 2016. Scoping review methods were used for their ability to explore broad research questions and interpret large amounts of material from various forms of data and research, while providing an important first step in synthesizing a complex body of research that can be used to guide the direction of future research [26, 27].

**Fig 1:**
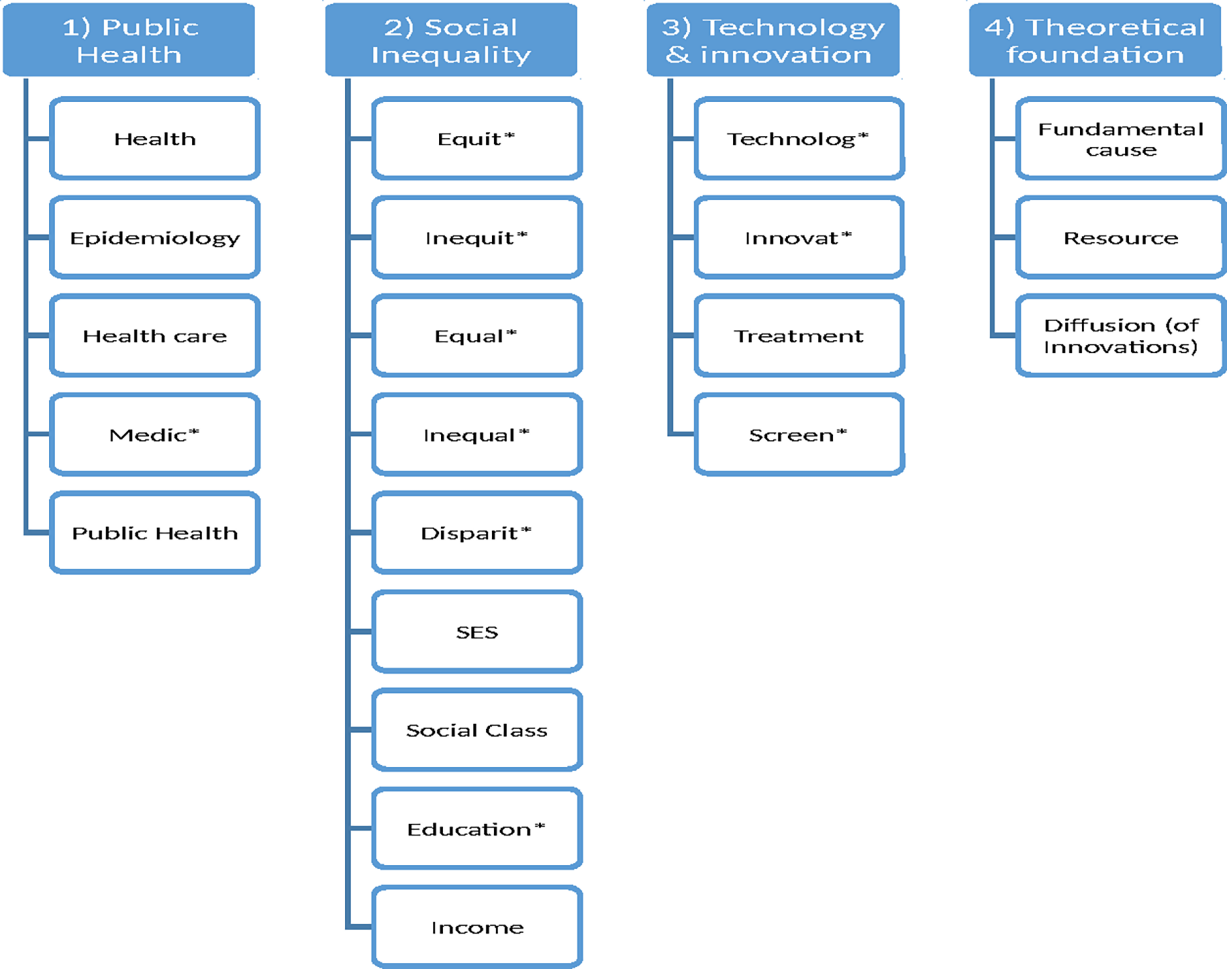
Search terms and their categorization into overarching themes.

Search terms were categorized into four categories (“public health,” “social inequality,” “technology and innovation,” “theoretical foundation”) in order to provide additional organization when combining terms during the search process (Fig 1). Only peer-reviewed studies based on original data analysis were included in this study, as interest was focused on collecting empirical analyses. A full overview of inclusion/exclusion criteria can be found in Table 1.

**Table 1:** Inclusion/Exclusion criteria.

Inclusion Criteria	Exclusion Criteria
English Language	Before 1996
Peer-reviewed original study or review, based on an original data analysis	Focus on health services or health care without specific focus on technology and inequalities
Addresses inequalities in health outcomes (also called health disparities, inequalities in health, health inequity, equity in health, etc.)	Innovations without a technological component or technologies with only a “software” component (such as new knowledge or cultural ideas)
Comparison of social groups/classes (i.e. low-income vs high income; rural vs urban; low educated vs high educated; white vs. Hispanic; etc.) or specific focus on a disadvantaged population.	Editorial, commentary, letters to the editor, columns, opinions, viewpoints, or similar
Specifically addresses technology (must include a “hardware” component, such as a tool or instrument)	
Explicit and identifiable application of *innovative* technology (new technology, or old technology used in a new way)	

The initial search process was performed by two research librarians with expertise in the use of literature databases. Extensive testing of the search strings was performed before the search process. To reduce the number of irrelevant hits and increase accuracy of the searches, a proximity operator was used as well as custom search strings for each database. Rationale and search strings for each individual database can be found in Table 2.

**Table 2:** Database rational and search strings.

Database	Rationale	Search string
Medline	As Medline is predominantly medically focused, a more permissive search string was used in order to open for a greater inclusion of medical studies focused on technology.	(health* OR epidemiology OR "health care" OR medic* OR "public health") adj5 (equit* OR inequit* OR equal* OR inequal* OR disparit* OR SES OR "social class" OR education* OR income) adj5 (technolog* OR innovat* OR treatment OR screen) adj5 ("fundamental cause*" OR resource OR diffusion OR innovation*)
Scopus	A stricter proximity search was used with Scopus. This was done to force the search to consider relevant words together.	(health OR epidemiology OR "health care" OR medic* OR "public health”) W/5 (equit* OR inequit* OR equal* OR inequal* OR disparit* OR ses OR "social clas*" OR education* OR income) AND (technolog* OR innovat* OR treatment OR screen*) AND ("fundamental cause" OR resource OR diffusion W/1 innovation*)
ISI Web of Science	Same as Scopus	(health OR epidemiology OR "health care" OR medic* OR "public health") near/5 (equit* OR inequit* OR equal* OR inequal* OR disparit* OR ses OR "social clas*" OR education* OR income) AND (technolog* OR innovat* OR treatment OR screen) AND ("fundamental cause" OR resource OR diffusion near/1 innovation*)

**Fig 2:**
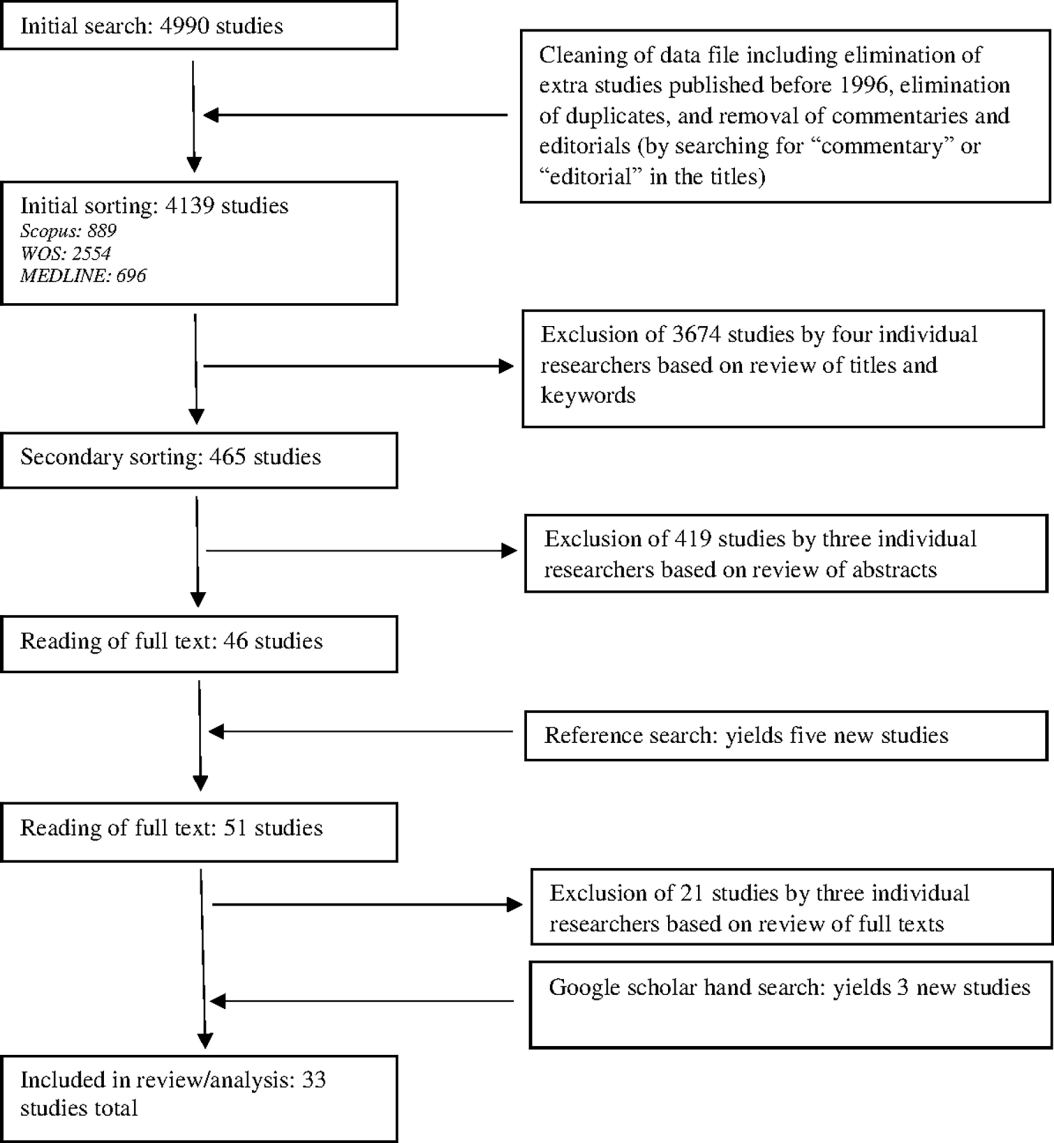
The sorting process.

The initial search resulted in a total of 4139 studies, after cleaning of the original data file. After sorting the dataset alphabetically by study title, the entire dataset was divided into four equal subsets. Each subset was then sorted independently by two individual researchers. Studies deemed relevant by both researchers automatically advanced to secondary sorting. A third researcher, who had not previously worked with the respective subset, then sorted those studies deemed relevant by only one of the two original researchers. Studies deemed relevant by the third researcher also advanced to secondary screening. All relevant studies from the initial screening process were then combined into a single dataset (465 studies) for use during the secondary screening process. During the secondary screening process, three individual researchers independently sorted all studies deemed relevant from the initial sorting process using abstracts (if abstracts were not present, results and conclusion sections were used to determine relevance). Only studies deemed relevant by all three researchers advanced to the final sorting process. In the final sorting process, three individual researchers independently read full texts of all included studies. Studies deemed relevant by all three researchers automatically advanced to the data extraction process, while studies deemed irrelevant by all three researchers were automatically excluded. Studies with inconsistent evaluations were discussed by all three researchers until agreement for inclusion or exclusion was met. The resultant studies from this multi-stage systematic sorting process were included in the data extraction process and presented in our results section (Fig 2). The inclusion/exclusion criteria was strictly applied at each stage of the sorting process and articles were excluded if deemed by multiple researchers to meet exclusion criteria based on title and keywords (stage 1), abstracts (stage 2), or full text (stage 3).

Data extraction was facilitated by the use of a data extraction form designed using procedures outlined by Armstrong et al. [25]. A data extraction form was used to systematically extract information relevant to the aims of this study as well as standard descriptive information. Along with standard title, author and year of publication information, categories included: study location; geographical level (local, regional, national or international); study population; methods used; specific illness addressed; technological innovation addressed/measured; method of implementation for the addressed technology; definition/measurement of social class/inequality; theoretical perspectives; main outcome measures (including health outcomes); overview of main results and conclusions. All full texts were read and analyzed by three individual researchers and individual data extraction forms were then merged into a single, unifying document used for the interpretation and presentation of results. Following typical scoping review methods, methodological quality of the included articles was not assessed systematically, however only peer-reviewed articles were included in our review process [24, 25, 28]. The lack of a systematic analysis of methodological quality is both a weakness and a strength of scoping review techniques. Although it is difficult for a scoping review to draw conclusions based on the quality of the included studies, the strength of a scoping review is in its ability to condense large amounts of material and guide the direction of future research including more comprehensive analyses of the quality of relevant methods [27, 28]. Assurance of methodological quality throughout the search, sorting and extraction processes in the current study however was protected using a systematic design based on a dynamic, reflexive examination process whereby multiple researchers, working at each stage of the process independently, regularly compared results and met to discuss, and reach agreement on, discrepancies [24, 27].

## Results and discussion

### Overview of included studies

An overview of included studies is offered in Table 3. An overview of excerpts from selected studies representing the formation of the narrative presented in the results and discussion section can be found as a table in supporting information (S1 Table. Forming the narrative–representative excerpts from selected studies). Data from the studies included in our results was most often collected using purely quantitative methods (N = 28), with some articles choosing to use mixed methods (N = 2) or qualitative methods (N = 3). Data collection varied widely between studies, with some studies addressing national populations, while others collected data at the hospital level or individual level. Of the studies addressing a specific illness (N = 18), approximately half of these addressed either HIV or blood/heart related illnesses. Of the technologies addressed by included studies, information/communication technologies (electronic health records and internet portals, e-health, internet-based social networks) and medical services technologies (prescription drugs, medical imaging, and diagnostic and treatment tools) dominated. Measurements of social position and inequality were relatively consistent with commonly used socio-economic variables, varying between income (or GDP in country comparisons), education, and employment status, in addition to geographical location, age, gender, and race/ethnicity. Outcome measures varied widely, however most studies were interested in investigating factors influencing the access, distribution, and/or use of specific technologies by various social groups (for example individual behaviors, facilitators and/or barriers). Some studies, however, addressed consequences associated with poor or limited access to these technologies, including related morbidity and/or mortality.

**Table 3:** Overview of included studies.

Authors	Country	Study population	Technological innovation measured or addressed	Social class/inequality variable	Main outcome measure(s)
Baum, Newman, & Biedrzycki (2014)	Australia	55 individuals located in areas with low SES	Information and communication technologies (ICT)	Race/ethnicity and socioeconomic status	Access and use of ICT
Bekelis, Missios & Labropoulos (2014)	United States	Patients undergoing any neurosurgical procedure 2005–2010	Cerebral aneurysm coiling	State/region, median income based on zip code	Average risk adjusted intensity of neurosurgical care and average coiling rate per state per year
Butler, Harootunian, & Johnson (2013)	United States	Physicians serving Medicaid and non-Medicaid patients in Arizona.	Electronic health records (EHR)	Insurance status	EHR access and use by general practitioners
Chang & Lauderdale (2009)	United States	Adults aged 20 and over from NHANES II, III, and continuous surveys.	Statin (HMG-CoA reductase inhibitors)	Socio-economic status by income	Income gradients for cholesterol levels over time
Cheng et al. (2012)	United States	Veterans hospitalized with ischemic stroke	Carotid artery imaging	Race/ethnicity	Receipt of carotid artery imaging; race of the patient and minority-serving status of the hospital
Choi & DiNitto (2013)	United States	Low-income homebound adults	Internet based information technology	Age and income	Internet use, eHealth literacy, attitudes toward computer/internet use
Eddens et al. (2009)	United States	Cancer survivors	Internet/e-Health	Race/ethnicity	Characteristics of cancer survivors, cancer type, form of communication, website characteristics
Ferris et al. (2006)	United States	Adults (under 60) and children with asthma.	Meter dose inhaler	Race/ethnicity and age	Use of meter dose inhalers Insurance status Physician visits and reason for visit
Glied & Lleras-Muney (2008)	United States	Persons diagnosed with cancer	Drug approvals by number of active ingredients approved by FDA	Education	Mortality and drug approvals
Goel et al. (2011)	United States	Patients from an urban, academic primary care practice	Patient health portals	Race/ethnicity, age, gender, education, income	Enrollment in the patient portal, Solicitation of provider advice among enrollees, Requests for medication refills among enrollees.
Goldman & Lakdawalla (2005)	United States	HIV positive, aged 18+ who made at least one visit to clinic in 1996; Men and women aged 28–59 in 1948 residing in Framingham, Mass.	Highly Active Antiretroviral Therapy; beta-blockers	Education	Exposure to drug and health status before and after introduction of technology
Gonzales, Ems, & Suri (2016)	United States	Adults from low-income groups and staff of health care organizations	Cell phones/m-Health	Income	Experiences and challenges to using cell phones and disconnection, as well as related challenges to access healthcare and other social services.
Groeneveld, Laufer, & Garber (2005)	United States	Elderly (over 65) Medicare beneficiaries	Various "emerging" technologies: aortic valve replacement, internal mammary artery coronary bypass grafting, dual-chamber pacemaker implant, vena cava interruption, and lumbar/lumbosacral spinal fusion	Race/ethnicity	Procedure rates using emerging technologies by race
Han, et al. (2010)	Australia	General population with at least one diagnosed chronic medical condition	Information and communication technologies	Socio-economic status	Internet accessibility, socio-economic status by geographical area, prevalence of chronic disease
He, Yu, & Chen (2013)	China	Random sample of 71 hospitals from four sites	CT and MRI scanners	GDP at a regional level	Gini coefficient (equity), distribution of CT and MRI, characteristics of CT and MRI machines
Hing & Burt (2009)	United States	Non-federal office-based primary care physicians or providers (PCP)	Electronic health records (EHR)	Payment source; race/ethnicity; median household income	Likelihood of PCPs using EHR
Horvitz-Lennon, Alegría, & Normand (2012)	United States	Medicaid beneficiaries with schizophrenia who had filled at least 1 antipsychotic prescription during the study period	Long-acting injectable formulation of the atypical antipsychotic risperidone (LAIR)	Race/ethnicity and geographic location	Use of LAIR
Kontos, Emmons, Puleo, & Viswanath (2010)	United States	Representative sample of US adults	Internet: social networking sites (SNS)	Race/ethnicity and socioeconomic position	Internet access and SNS use
Korda, Clements, & Dixon (2011)	Australia	Patients (≥35 years of age) with a principal or co-diagnosis of acute myocardial infarction (AMI), and with no previous admissions for AMI, between 1989 & 2003.	Coronary procedures: angiography, angioplasty and coronary artery bypass surgery	Socio-economic status by SIEFA index of disadvantage	Receipt of a coronary procedure
Lang & Mertes (2011)	Europe	24 EU member states	E-health	Economic variables (GDP per capita, ICT market value, Broadband access in enterprises)	Effect of various economic, healthcare, and political variables on the implementation of e-health applications
Loureiro et al. (2007)	Brazil	Brazilian states	MRI, computerized tomography, and dialysis machines	Regional socio-economic status by GDP per capita	Distribution of access; number (surplus/deficit) of machines; public vs. private sector differences
Newhouse et al. (2015)	Many	Citizens 16–74 years of age who had used the internet in previous 3 months	Internet based information technology/e-mail	Geographical; education; gender; employment status	Frequency of sending emails to health personnel
Newman, Biedrzycki, & Baum (2012)	Australia	Residents from lower income and disadvantaged backgrounds in South Australia	Information and communication technologies (ICT)	Socioeconomic status	Access, usage and perceived facilitators and barriers to ICT
Ohl et al. (2013)	United States	Veterans in care for HIV infection	Combination antiretroviral therapy (cART)/raltegravir	Geographic (urban/rural); race/ethnicity; age/gender	Raltegravir adoption
Ohlsson, Chaix, & Merlo (2009)	Sweden	Individuals in Skåne region who were issued at least one prescription for statins between July and December 2005	Rosuvastatin (prescription statin)	Socio-economic status	Factors related to outpatient health care practice; physicians’ propensity to prescribe rosuvastatin
Perez et al. (2016)	United States	Participants 21 to 35 years of age, had searched the Internet for health information within the past 12 months, and reported at least one barrier to health care services access.	Internet based IT	Education; recruitment from sites offering/not offering social services	Internet search behavior, strategies and processes
Polonijo & Carpiano (2013)	United States	Adolescent girls (age 13–17) and their parents/guardians	HPV vaccine (cervarix/gardasil)	Socio-economic status; race/ethnicity	Parental knowledge of the vaccine; health professional's recommendation of HPV vaccination; actual uptake, and finishing, of the vaccine
Rubin, Colen, & Link (2010)	United States	HIV positive black and white men and women between the age of 15 to 64	Highly active antiretroviral therapy	Socio-economic status; race/ethnicity	HIV/AIDS mortality before and after the introduction of highly active antiretroviral therapy
Slade & Anderson (2001)	Many	OECD countries between 1975–1995	MRI machines, CT scanners, kidney transplants, liver transplants, and hemodialysis patients	GDP per capita	Availability and utilization of technology
Stanley, DeLia, & Cantor (2007)	United States	Individuals at risk for sudden cardiac death (SCD)	Implantable cardioverter defibrillator	Race/ethnicity	ICD use and utilization
Wang et al. (2010)	Taiwan	Osteoarthritis patients (≥60 years of age) who had undertaken at least one outpatient visit for osteoarthritis	NSAIDs	Income	Treatment incidence
Woolf et al. (2007)	United States	General population (adults 18–64 years of age)	General technological innovations	Education	Age-adjusted mortality
Zibrik et al. (2015)	Canada	Participants from Chinese and Punjabi public health education events	E-health: online tools for health education, communication and self-management	Ethnicity/immigrant status, age, gender, income, and education	e-health literacy

### Addressing classification and measurement challenges: Towards a more precise terminology

#### Social inequality

All variables used in included studies to address, define and measure social position acknowledge that these variables represent various social groups, or classes, that live in relative advantage/disadvantage to one another. These variables can be divided into three distinct approaches. The first approach is characterized by a distinction between selected social groups based on fixed (or ascriptive) factors. These studies use age, gender and/or race/ethnicity to define and measure differences between social groups. The second approach is characterized by social position determining an individual’s control of various flexible resources that are to a relative degree amendable [15]. These studies generally stratify social position based on socio-economic variables such as education, income, and insurance or employment status. Unlike the two aforementioned approaches, the third approach is distinguished by the characteristics of place [29]. These studies use geographic location as a measure of social stratification, often defined as (but not limited to) a distinction between rural and urban settings.

These distinct approaches are similarly used to investigate social inequalities, however it is possible to question whether these distinct approaches can be used interchangeably to understand variations in the distribution of population health and innovative health technologies. Although SES may, for example, include various measures such as education, income, and occupational status, used alone or in combinations, one could question whether the mechanisms connecting education to health and technology are the same as the mechanisms connecting occupation or insurance status to health and technology. In relevant literature, such reflections are by and large missing, and very different measures of social position are often treated and interpreted similarly, which may affect the applicability and usability of results [30]. The implications of choosing one approach over another may have consequences on both theoretical and practical understandings of the specific social factors that influence access and use of innovative health technologies. In the studies included in our analysis, it is possible to observe variations in measured inequality based on chosen variables. The variation in results from these studies illustrate that whether or not inequalities in access and use of innovative health technologies are observable are dependent on the approach used to measure these inequalities and that common measures of social inequality in health cannot be used uncritically.

Our findings, however, may suggest that variations in measurement techniques are, in part, rooted in cultural or scientific traditions. It is interesting to note, for example, that although many of the studies from North America and Australia used a variety of approaches to measuring social inequality, race/ethnicity was often included. Race/ethnicity was, however, never included as a variable in collected studies originating from European, Asian, or South American countries, which instead favored the use of various measures of socio-economic status, such as income or education. Our results do not provide a clear explanation to this finding, but one may question whether this is due purely to availability of data or to cultural and historical factors, where race and ethnicity are more strongly associated with social stratification and class positions in North America and Australia [31, 32]. Regardless, the previous findings raise important questions regarding the extent to which social inequalities in access and use of innovative health technologies are dependent on the approach used to measure and define social groups, which must be critically addressed in future research.

#### Innovative health technologies

**Fig 3:**
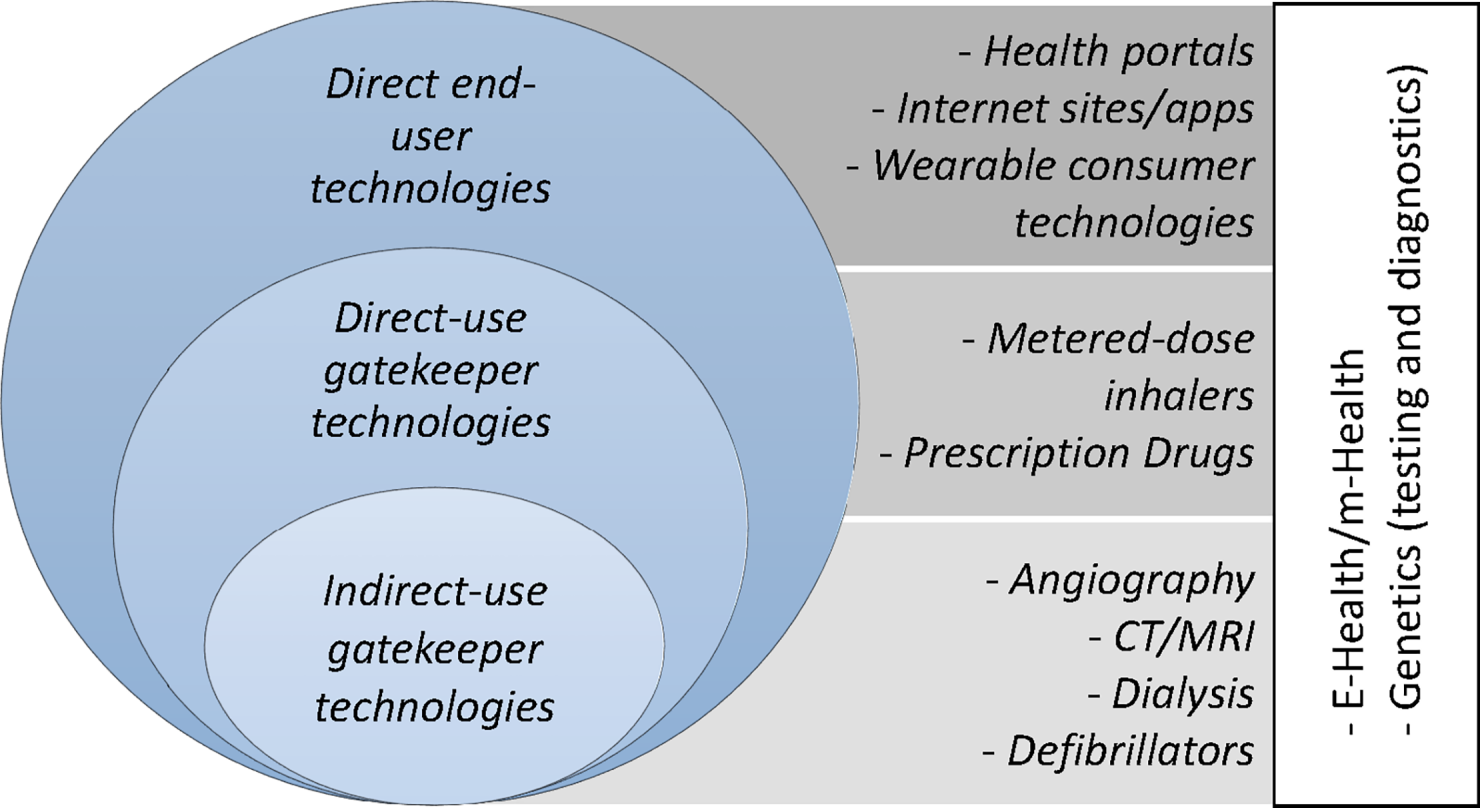
Classification of technologies.

Although it is possible to broadly categorize technologies in included studies by type, a potentially more informative method of categorizing these technologies from a social inequalities in health context is by variations in access and use. Using an approach similar to those presented by Cotterman and Kumar [33], and a focus on level of perceived end-user control, it is possible to propose a division of technologies into three main categories (see Fig 3): technologies accessed and used directly by the end user (type 1 or *direct end-user technologies*), technologies used by the end user but accessed through someone other than the end user (type 2, or *direct-use gatekeeper technologies*), and technologies accessed and used by someone other than the end user (type 3, or *indirect-use gatekeeper technologies*). In this case “end user” is defined as the individual, or group of individuals, for which the technology is developed. End users generally do not include individuals who develop, operate, or distribute these technologies, unless these individuals are also end users (the operator of a direct end-user technology, for example, is also the end user). As the name implies, “gatekeepers,” in this case, are individuals that guard access and eventual use of technologies by end users [13].

In the case of indirect-use gatekeeper technologies (type 3) and direct-use gatekeeper technologies (type 2), end-users are dependent on gatekeepers in order to gain access to these technologies. Korda et al. [20], for example, investigated the use of a number of coronary procedure technologies dependent on the expertise of health care personnel in which end users have very little direct control over the use and administration of these types of technologies [20, 34]. The technology examined by Rubin et al. [35] (highly-active antiretroviral therapy) differed in that, although access is dependent on a physician, use of the technology is significantly dependent on behavior by the end-user. Results by both Korda et al. [20] and Rubin et al. [35] demonstrate that, after the initial adoption of these technologies, social inequalities in health grew, regardless of whether the use of these technologies was dependent on end user behavior and, furthermore, regardless of the fact that these technologies must be accessed by way of trained health personnel. Results by Korda et al. [20] however also suggest that these inequalities may decline over time, as the adoption by lower SES groups increases.

“The SES inequalities in diffusion observed for angiography and CABG are consistent with the lag in diffusion/inverse inequality hypothesis–for both these procedures, rates peaked earlier in the higher SES patients than the lower SES patients resulting in inequalities, which then disappeared over time…”[20, 36]

Similar findings are corroborated by He et al. [37], Ohl et al. [38], and Stanley et al. [39]. Moreover, results by Goldman and Lakdawalla [40] demonstrate that complicated treatment regimens increase social inequalities in health while simplifying treatment regimens reduce inequalities, illustrating the dynamic complexity of the relationship between access and use of innovative technologies and variations in social inequalities in health.

“Simply by improving the productivity of healthcare, new technologies can widen disparities across socioeconomic groups. However, new treatments that simplify the production of health and reduce the importance of patient effort work in the opposite direction…complex new treatments for HIV appear to have increased disparities among HIV+ individuals, while pharmaceutical breakthroughs in the treatment of hypertension made self-management less important and coincided with a contraction in disparities…”[40]

Nevertheless, the results highlighted above suggest that SES influences variations in the use of innovative technologies by end users even when access is dependent on a “gatekeeper”.

Direct end-user technologies (type 1), contrary to direct-use gatekeeper (type 2) and indirect-use gatekeeper technologies (type 3), are directly accessed and used by end users. The access and use of these technologies is assumed largely dependent on individual agency, or in other words, the assumption that individuals are equally able to consciously make decisions to access or use these technologies for purposes of influencing health. However, the studies included in our results consistently demonstrated that access and use of these technologies was far from equal. Baum et al. [41], for example, demonstrated that low socio-economic groups have restricted access and use of digital information and communication technologies that, in turn, affect access to a range of social determinants of health, creating a vicious cycle of disadvantage and poorer health.

“The educational opportunities to acquire fundamental literacy also shape health literacy, which therefore in turn affects people's ability to improve their health status and health outcomes. This disadvantage is compounded because digital literacy is increasingly a pre-requisite for health service delivery and access to health information.”[41]

Gonzales et al. [42] indicate that access to technologies for disadvantaged groups is unstable, and can be regularly disrupted, suggesting that simply measuring access to technology adoption across socioeconomic groups ignores the possibility that unstable access–or unequal use–can have large consequences on social inequalities in health. Perez et al. [43] support these results, further demonstrating that purely having access to a particular technology does not guarantee equal use. In fact, an increase in social inequalities in health after the implementation of health technologies is often demonstrated by studies included in our results. Importantly, regardless of findings suggesting that these inequalities will decrease as access to these resources becomes more universal, results from included studies illustrate that access to resources does not necessarily eliminate the (re)production of social inequalities in health.

Unfortunately, our results do not clearly illustrate whether any one of the categories of technologies highlighted in included studies has the potential to influence social inequalities in health to a greater degree than another. Our findings do, however, illustrate a complex relationship, suggesting that the pathways and mechanisms through which inequalities increase or decrease over time vary depending on the factors that influence *both* access and use, as well as type, of these technologies. Furthermore, it was rare for studies included in our results to explicitly measure health outcomes related to the access or use of these technologies. Therefore, studies rarely addressed or investigated specific mechanisms or pathways linking health technology access and/or use to unique explanations of variations in health. Consequently, it is clear that more research is needed to further understand these complex mechanisms.

It is also clear that some important technologies are missing from the literature. The technologies addressed by studies included in our results focus predominantly on technologies designed and used in health care services. Included in this collection of technologies is a growing focus on the internet and internet-based tools, as the use of these technologies also become an integrated resource in health care services [3, 4, 42, 44–46]. However, as various researchers have highlighted in recent years, technologies that have the potential to greatly influence health and social inequalities in health are not limited to those found in health care services [3, 6, 7, 11]. These technologies include innovations used to monitor and control individual health, such as genome sequencing and lifestyle technologies (wearable devices and personal, digital applications, for example). It is, therefore, clear that future research investigate the potential implications of these types of innovative technologies on social inequalities in health.

### Discussing potential pathways: Conceptualizing access and use

The studies included in this article exhibit varying approaches for conceptualizing the relationship between innovative health technologies and social inequalities in health. Studies discussing a perspective grounded in individual access and adoption of these technologies [18, 20, 39, 47–49] often refer to the diffusion of innovations theory, which categorizes adopters of innovations based on individual characteristics related to social positioning [13]. These studies use this theory to establish that lower SES groups are slowest to adopt, and therefore benefit less from, innovative health technologies. However, as access to these technologies “diffuses” throughout the population, and lower SES groups begin to adopt, these inequalities begin to diminish and may potentially disappear [18, 20, 37–40, 47].

“Income gradients were positive in an era prior to statins, but became negative in the period subsequent to the advent and dissemination of statins. While the more advantaged were once more likely to have high levels of cholesterol and LDL, they are now definitively less likely. Additionally, exploratory analyses suggest that income is positively associated with statin use accounting for clinical need… While resources affect access to technologies, some technologies can also affect resources, lessening the productivity of various health inputs.”[18]

Although this perspective assumes that the unequal adoption of these technologies is relatively unavoidable, they argue that the extent to which these innovations influence social inequalities in health is subject to the rate at which these technologies diffuse.

Building on this explanation, a number of studies [18, 19, 35, 47, 50] draw attention to the fundamental cause theory, which suggests that individuals “deploy” flexible resources, “such as money, knowledge, power, prestige, and beneficial social connections…to avoid risks and adopt protective strategies” [15]. These studies use this theory to illustrate that innovative health technologies are accessed to a greater degree by individuals of higher social position.

“The SES–HIV/AIDS mortality association, although present in the pre HAART period, was greater in the peri-HAART period and greater still in the post HAART period, even when race and other factors were controlled…These findings are consistent with fundamental cause theory, which holds that when innovations render a disease more treatable, the benefits of such developments are not evenly distributed.”[35]

Explanations referring to the fundamental cause theory and the diffusion of innovations, however, often assume that as innovative health technologies become more evenly distributed–or adopted–across social strata, so too will their benefits.

The above perspectives are contrasted by studies presenting social inequalities more specifically as a consequence of variations in use of innovative health technologies. These discussions often refer to explanations grounded in theories related to health literacy [43, 44, 51, 52] or digital divide [41, 42, 46, 53–55]. While health literacy refers to an individual’s ability to assess, understand, and use information critical to using health services and making decisions regarding health [52], digital divide refers to variations in the use of digital technologies between social strata [54]. These studies suggest that, regardless of access, inequalities exist due to the characteristics of social position determining an individual’s proficiency in using innovative health technologies to benefit health. Perez et al. [43], for example, demonstrate that, regardless of access to internet-based tools, health information searching and processing strategies vary by SES, benefitting higher educated individuals.

“When confronted with a specific set of symptoms, higher-SES participants tended to use search strategies that branch out—the exploration of conditions they expect contribute to the symptoms and systematically exploring offshoots of that condition, such as related conditions or symptoms. Lower-SES participants used heuristics to prune the scope of their Internet search—i.e., heuristics to ignore or remove search topics believed to be superfluous to the condition.”[43]

Results by Zibrik et al. [52] and Newman et al. [55] illustrate the significance of socioeconomic and cultural factors influencing variations in the quality of use of innovative health technologies, favoring individuals of higher social position. These studies emphasize the experiences of individuals with innovative health technologies, demonstrating that variations in user experience as a result of social positioning has the potential to undermine the benefits assumed by universal access.

The above theories, however, seem to suggest that these inequalities are driven by the potential of social positioning to provide individuals with the ability to make conscious choices and “consume” these resources [56], assuming that these choices are made consciously and with motivated intent to improve health [23]. However, numerous studies included in our results highlight the importance of mechanisms at the institutional and political levels that may significantly influence the distribution, in access and use, of innovative health technologies across social strata [14, 34, 37, 45, 57–64]. Many of these studies demonstrate that patterns of adoption and use of innovative health technologies at the level of the health care institution may significantly influence the potential of these innovations to benefit the health of end users regardless of individual choice or intent.

“Patients admitted to non-minority-serving hospitals were more likely to receive carotid artery imaging than patients admitted to minority-serving hospitals…the predicted probabilities of receiving carotid artery imaging were similar between white patients and black patients at non-minority-serving hospitals…However, the predicted probabilities among white patients and black patients at minority-serving hospitals were both significantly lower than white patients at non-minority-serving hospitals.”[34]

Furthermore, a study by Lang and Mertes [62] demonstrated that the prevailing orientation of dominating political parties can influence how innovative health technologies are accessed and used at the State level, resulting in variations in the distribution of these resources. In a similar discussion, Han et al. [60] refer explicitly to the social determinants of health theory, which describes the unequal distribution of health as a result of socioeconomic conditions that are largely constructed by social policy [65], to stress the significance of a geographical patterning of health influencing variations in access and use of innovative health technologies.

Due to a focus on single technologies, however, many of the perspectives discussed above fail to address the potential influence that the rapid, uninterrupted development of new technologies may have on the reduction or (re)production of social inequalities in health. It could be suggested that the cumulative effects of multiple technologies adopted over time is itself a mechanism for (re)producing health disparities. In this case, potential mechanisms could be related to windfall benefits [13], which are benefits afforded by early adopters (high SES individuals) that accumulate over time, or Bourdieu’s theories of capital and symbolic violence [66], where the development and implementation of innovative technologies by high SES groups may reinforce social stratification. Baum et al. [41] demonstrate that Bourdieu’s social theories are a relevant addition to a discussion of innovative health technologies and social inequalities in health, drawing attention to the ways that innovative health technologies potentially influence the interaction of social, cultural, and economic capital to reproduce inequalities in health. They conclude that “some people are being caught in a vicious cycle whereby the inability to make beneficial use [of innovative health technologies] reinforces and amplifies existing disadvantage” [41].

The results of this study, therefore, seem to suggest that understanding the pathways through which various innovative health technologies reduce or (re)produce social inequalities in health is context dependent. Theories focused on the dependency of individual resources, such as fundamental cause theory, may therefore be most appropriate for understanding socially stratified variations in the access and use of direct end-user (type 1) technologies. Interestingly enough however, studies referring to these theories generally address direct and indirect-use gatekeeper (types 2 and 3) technologies, allowing one to question the merit of these explanations. Conversely, mechanisms at the institutional and political levels would thus seem most appropriate in explaining direct and indirect-use gatekeeper (types 2 and 3) technologies, where the advantages of these technologies are often poorly recognized by individuals of lower social status or where access is limited by gatekeepers (for example, political or institutional agents). In order to better understand social inequalities in health, these contextual variations draw attention to the need for critical distinctions between technologies based on how, and in what context, these various technologies are accessed and used. This may include a stronger focus on understanding the role of institutions and accompanying theories that explain complex mechanisms influencing the distribution of population health [1].

### Limitations

Some limitations not addressed earlier in this study are worth discussing. First, although the choice of search terms was purposefully broad and systematically identified using relevant literature, it is possible that the ability to collect relevant literature from a larger breadth of research fields and traditions could have been limited. This is due to the possibility that the researchers’ previous relation to the fields of sociology and health limit the familiarity, and therefore inclusion, of relevant terms or language used in the fields of technology and innovation. Second, the decision to exclude grey literature, including books, reports, etc., may have led to the exclusion of relevant literature, which could have possibly been used to widen or further support perspectives presented in the results. However, this choice was made with consideration for a purposeful selection of empirical, peer-reviewed studies using original data analyses. The goal here was to increase the probabilities of including relatively high quality research and excluding the possibility of grey material that is lower in quality and neither peer-reviewed nor includes original analyses. Furthermore, as grey literature includes reports and documents often drafted by order of political or special interest organizations, it is more difficult to assess underlying biases that would negatively bias our results. Third, the decision to exclude studies focused on treatment techniques within health services may have excluded some relevant literature. Very often, treatment techniques are dependent on the use of a specific technology. However, had the current study included literature focused on treatment techniques, without a specific focus on the technology used in this treatment, it would have been up to the authors to investigate whether or not each treatment technique included the use of an innovative technology, introducing bias as well as a very problematic assessment process. Furthermore, the inclusion of such studies would have shifted the focus of the current study from that of one focused on novel perspectives related to technology and public health to one focused on the relatively well established field of social inequalities in treatment and health services. The authors, therefore, felt that the inclusion of such studies was out of the scope of the current study and would have fundamentally transformed the current study’s aims and contribution to the scientific literature.

## Conclusions

This review was interested in systematically investigating existing literature that explores the influence of innovative technologies on social inequalities in health. The results of this study offer interesting perspectives worth consideration, with implications for further investigation of the influence of innovative health technologies on social inequalities in health. This study questions established scientific measures of social inequality, where various measurements (such as race/ethnicity, income, education, geography, etc.) are often used interchangeably to investigate variations in access and use of innovative health technologies. Results illustrate that the choice of measurement has the potential to bias findings and, therefore, significantly influence the understanding of complex relationships between innovative health technologies and social inequalities in health. Furthermore, this study proposes that a social inequalities perspective may benefit from an understanding, and differentiation, of technologies based on how these technologies are accessed and used by end users. Factors and mechanisms that influence access, for example, may differ from factors and mechanisms that influence use. It is clear that it is not enough to solely focus on the factors and mechanisms that influence unequal access and therefore ignore how variations in use importantly shape social inequalities in health. It is, moreover, not enough to focus attention solely on health care services technologies but, importantly, to investigate emerging technologies in lifestyle health, genomics, and the increased use of personalized devices in health. Furthermore, a deeper understanding of social inequalities in health and innovative health technologies is dependent on distinguishing between a perspective focused on individual resource use, which often draws a questionable causal relationships between SES, technology access/use, and health outcomes, and a perspective focused on mechanisms that are more dependent on social and institutional structure than on individual agency. Although the studies included in our results generally suggest that the implementation and adoption of new technologies (re)produce SES and class-based social inequalities in health, some results indicate that these technologies can, in fact, reduce inequalities over time. Additional research, based on the findings discussed in this study, are needed, however, to reliably establish these conclusions. As much of the current research is dominated by the use of quantitative methods of social epidemiology, additional research may benefit from an increased use of qualitative, sociological methods in order to further investigate mechanisms and pathways leading to the (re)production of social inequalities in health as a result of innovative technologies [8, 30]. It is, nevertheless, becoming increasingly important to investigate the social implications and consequences of a society increasingly influenced by technological innovations, including the ways in which these technologies may influence the unequal distribution of health as a human right.

## Supporting information

S1 Table Forming the narrative–representative excerpts from selected studies. (DOCX)

S1 File Original data file. (TXT)

S1 PRISMA Checklist (DOC)
