# Supplementary material for: Innovative technologies and social inequalities in health: A scoping review of the literature
Source: PLoS One. 2018 Apr 3;13(4):e0195447. doi: 10.1371/journal.pone.0195447 (PMC5882163; doi:10.1371/journal.pone.0195447)
Supplement: S1 Table — (DOCX) [file pone.0195447.s001.docx]

| **Study title** | **Category/Importance^†^** | **Excerpt** |
| --- | --- | --- |
| Korda, Clements, & Dixon (2011) | Type 3 technology. Technologies accessed and used by way of a gatekeeper present inequalities following the diffusion of innovations theory. Here when SES is used to measure inequalities. | “The SES inequalities in diffusion observed for angiography and CABG are consistent with the lag in diffusion/inverse inequality hypothesis – for both these procedures, rates peaked earlier in the higher SES patients than the lower SES patients resulting in inequalities, which then disappeared over time…” |
| Rubin, Colen, & Link (2010) | Type 2 technology. Technologies accessed by way of a gatekeeper but used by end-users present inequalities following fundamental cause theory (and similar to diffusion of innovations theory). Here when race and SES are used to measure inequalities. | “The SES–HIV/AIDS mortality association, although present in the pre HAART period, was greater in the peri-HAART period and greater still in the post HAART period, even when race and other factors were controlled…These findings are consistent with fundamental cause theory, which holds that when innovations render a disease more treatable, the benefits of such developments are not evenly distributed.” |
| Chang & Lauderdale (2009) | Type 2 technology. Similar conclusions and trends found in Rubin, Colen, & Link (2010). But here also presenting results highlighting the importance of social conditions influencing the length and strength of the diffusion process. Here when income is used to measure inequalities. | “Income gradients were positive in an era prior to statins, but became negative in the period subsequent to the advent and dissemination of statins. While the more advantaged were once more likely to have high levels of cholesterol and LDL, they are now definitively less likely. Additionally, exploratory analyses suggest that income is positively associated with statin use accounting for clinical need… While resources affect access to technologies, some technologies can also affect resources, lessening the productivity of various health inputs.” |
| Ohl et al. (2013) | Type 2 technology. Although increases in inequality followed adoption curves (diffusion of innovations theory), these inequalities reduced over time. Here when inequalities are measured using geographical variables. | “Urban veterans were more likely than rural to be early adopters of raltegravir, although absolute differences in adoption had slightly narrowed by two years after raltegravir approval.” |
| Stanley, DeLia, & Cantor (2007) | Type 3 technology presenting similar trends as Ohl et al (2013). Here also when race and SES are used to measure inequalities. | “This study finds that the use of ICD therapy among  black adults (ages .18) at risk for SCD was consistently lower relative to nonblack, at-risk adults from 1996-2001… Rates of ICD use grew for blacks and others between the early (1996-1998) and later (1999-2001) years of the study, with faster growth occurring within the black population.” |
| Goldman & Lakdawalla (2005) | Various type 2 technologies. Illustrate both increases and decreases in disparities based on the way in which the technology is used by end-users. Here with education as a measure of inequality. | “Simply by improving the productivity of healthcare, new technologies can widen disparities across socioeconomic groups. However, new treatments that simplify the production of health and reduce the importance of patient effort work in the opposite direction…complex new treatments for HIV appear to have increased disparities among HIV+ individuals, while pharmaceutical breakthroughs in the treatment of hypertension made self-management less important and coincided with a contraction in disparities…” |
| Baum, Newman, & Biedrzycki (2014) | Type 1 technologies. Individual socio-economic resources can have a significant impact on the way technologies are used by end-users even when access is secured. Here with race and SES as a measure of inequality. | “The educational opportunities to acquire fundamental literacy also shape health literacy, which therefore in turn affects people's ability to improve their health status and health outcomes. This disadvantage is compounded because digital literacy is increasingly a pre-requisite for health service delivery and access to health information.” |
| Newman, Biedrzycki, & Baum (2012) | Type 1 technologies. Similar trends to those presented in Baum, Newman, & Biedrzycki (2014). Here with SES as a measure of inequality. | “Despite the almost universal ownership of mobile phones, many in our study perceived they had insufficient income to make mobile calls, limiting mobiles as a reliable communication option. For example, until 1800 numbers become free from mobile phones, services could consider how to support mobile freecalling such that a 1800 Smoking Quitline, for example, is affordable for the mobile-only population. Otherwise, such strategies will disproportionately affect lower SES groups, who are more likely to smoke.” |
| Gonzales, Ems, & Suri (2016) | Type 1 technology. Similar trends to those presented in Baum, Newman, & Biedrzycki (2014) and Newman, Biedrzycki, & Baum (2012). Here with income as a measure of inequality. | “These findings support technology maintenance predictions that low-income populations struggle to maintain digital access after ownership and public availability of new technologies are realized.” |
| Perez et al. (2016) | Type 1 technology. Similar trends to those presented in Baum, Newman, & Biedrzycki (2014), Newman, Biedrzycki, & Baum (2012) and Gonzales, Ems, & Suri (2016). Here with education as a measure of inequality. | “When confronted with a specific set of symptoms,  higher-SES participants tended to use search strategies that branch out—the exploration of conditions they expect contribute to the symptoms and systematically exploring offshoots of that condition, such as related conditions or symptoms. Lower-SES participants used heuristics to prune the scope of their Internet search—i.e., heuristics to ignore or remove search topics believed to be superfluous to the condition.” |
| Glied & Lleras-Muney (2008) | Type 2 technologies. Innovative technologies increase education-based social inequalities, supporting fundamental cause theory and diffusion of innovations theory. | “Studies of technological diffusion in other contexts consistently point to education as a factor that increases the diffusion rate… We relate education gradients in mortality to two measures of health related innovation and show that education gradients become larger for diseases with more innovation. We find that the pattern holds for mortality rates from all causes and for cancer mortality conditional on diagnosis.” |
| Zibrik et al. (2015) | Type 1 technologies. Perceived accessibility may mask the effects of (re)producing inequalities in health through unequal adoption processes. | “…eHealth literacy and eHealth uptake are lowest in immigrant groups, ethnic minorities and seniors… While important, accessibility alone does not guarantee utilization; it is essential to consider the way in which culture, social determinants, language, interest and attitudes factor into optimal eHealth resource use.” |
| Bekelis, Missios & Labropoulos (2014) | Type 3 technology. Personal financial resources act as a fundamental cause of increasing inequalities in health by way of unequal adoption and diffusion. | “At the patient level we demonstrated that increasing income was related to a higher rate of [the novel technology of cerebral aneurysm] coiling.” |
| Cheng et al. (2012) | Type 3 technology. Race (and SES) can have a direct effect on the healthcare resources an individual has access to, which then affect the access to innovative technologies through gatekeepers at these institutions. | “Patients admitted to non-minority-serving hospitals were more likely to receive carotid artery imaging than patients admitted to minority-serving hospitals…the predicted probabilities of receiving carotid artery imaging were similar between white patients and black patients at non-minority-serving hospitals…However, the predicted probabilities among white patients and black patients at minority-serving hospitals were both significantly lower than white patients at non-minority-serving hospitals.” |

^†^More information on the specific technologies found in these studies can be found in Table 3 under “Technological innovation measured or addressed.”
